# Seasonal Carbonate Chemistry Covariation with Temperature, Oxygen, and Salinity in a Fjord Estuary: Implications for the Design of Ocean Acidification Experiments

**DOI:** 10.1371/journal.pone.0089619

**Published:** 2014-02-19

**Authors:** Jonathan C. P. Reum, Simone R. Alin, Richard A. Feely, Jan Newton, Mark Warner, Paul McElhany

**Affiliations:** 1 Conservation Biology Division, Northwest Fisheries Science Center, National Marine Fisheries Service, National Oceanic and Atmospheric Administration, Seattle, Washington, United States of America; 2 Pacific Marine Environmental Laboratory, National Oceanic and Atmospheric Administration, Seattle, Washington, United States of America; 3 Applied Physics Laboratory, University of Washington, Seattle, Washington, United States of America; 4 School of Oceanography, University of Washington, Seattle, Washington, United States of America; University of Vigo, Spain

## Abstract

Carbonate chemistry variability is often poorly characterized in coastal regions and patterns of covariation with other biologically important variables such as temperature, oxygen concentration, and salinity are rarely evaluated. This absence of information hampers the design and interpretation of ocean acidification experiments that aim to characterize biological responses to future pCO_2_ levels relative to contemporary conditions. Here, we analyzed a large carbonate chemistry data set from Puget Sound, a fjord estuary on the U.S. west coast, and included measurements from three seasons (winter, summer, and fall). pCO_2_ exceeded the 2008–2011 mean atmospheric level (392 µatm) at all depths and seasons sampled except for the near-surface waters (< 10 m) in the summer. Further, undersaturated conditions with respect to the biogenic carbonate mineral aragonite were widespread (Ω_ar_<1). We show that pCO_2_ values were relatively uniform throughout the water column and across regions in winter, enriched in subsurface waters in summer, and in the fall some values exceeded 2500 µatm in near-surface waters. Carbonate chemistry covaried to differing levels with temperature and oxygen depending primarily on season and secondarily on region. Salinity, which varied little (27 to 31), was weakly correlated with carbonate chemistry. We illustrate potential high-frequency changes in carbonate chemistry, temperature, and oxygen conditions experienced simultaneously by organisms in Puget Sound that undergo diel vertical migrations under present-day conditions. We used simple calculations to estimate future pCO_2_ and Ω_ar_ values experienced by diel vertical migrators based on an increase in atmospheric CO_2_. Given the potential for non-linear interactions between pCO_2_ and other abiotic variables on physiological and ecological processes, our results provide a basis for identifying control conditions in ocean acidification experiments for this region, but also highlight the wide range of carbonate chemistry conditions organisms may currently experience in this and similar coastal ecosystems.

## Introduction

Global surface ocean pH levels have decreased by approximately 0.1 units since the Industrial Revolution and will potentially decline an additional 0.3 to 0.4 units over the next century due to anthropogenic CO_2_ emissions [1]–[4]. This change in ocean chemistry, termed “ocean acidification” (OA), results in a reduction in the saturation state of carbonate minerals such as calcite and aragonite, elevates pCO_2_, and lowers pH [5]. A growing body of research indicates that such changes may impact a diversity of species, with some organisms benefiting from increased pCO_2_ availability (e.g., photosynthesizers), while others (e.g., echinoderms, molluscs, some fishes) may be adversely impacted by enhanced shell dissolution rates, reductions in metabolic scope, or neurological impairment [2], [6]–[8]. A major challenge to inferring the ecological implications of these studies, however, is an incomplete understanding of present-day patterns of carbonate chemistry variability, particularly in coastal habitats [9]-[14].

In continental shelf and estuarine systems, complex physical processes and high rates of biological production act to perturb pCO_2_ levels away from conditions under air-sea gas equilibrium [9] and may exhibit considerable variability over interannual, seasonal, and daily time scales [10], [14]–[17]. In contrast, researchers studying organisms from these systems often use IPCC projections of global average surface ocean carbonate chemistry conditions that correspond to assumptions of air-sea gas equilibrium to set control and experimental carbonate chemistry conditions [11], [18]. Recognition of the potential disparity between conditions considered “controls” in OA experiments and actual carbonate chemistry conditions experienced by organisms in the habitats they occupy has been highlighted in a suite of recent publications [9], [10], [18]–[20]. Consequently, there is increasing awareness that researchers should attempt to simulate control conditions that correspond to carbonate chemistry conditions experienced by organisms in natural habitats and interpret experimental outcomes accordingly [21], [22].

However, largely missing from this discussion is the observation that carbonate chemistry commonly covaries with other biologically relevant variables including temperature and oxygen.

In some regions, the strength of these associations has permitted the development of semi-mechanistic predictive models of carbonate chemistry in coastal environments based on field measurements of temperature and oxygen [15], [23]. Yet for researchers focused on laboratory OA experiments, these relationships are rarely incorporated into experimental designs. This oversight poses important potential drawbacks. Foremost, if carbonate chemistry strongly covaries with temperature and oxygen, researchers risk running experiments with control water characteristics that are atypical of the habitat that a focal organism / life stage occupies. Physiological parameters including aerobic capacity and metabolic scope (the amount of energy that can be allocated to activities beyond those for basic existence) are strongly influenced by temperature, and adversely impacted by reductions in ambient oxygen availability or increases in CO_2_ concentrations [24]. Under extreme warm and cool temperatures defining the upper and lower boundaries of the thermal window an organism survives within, the negative effects of oxygen scarcity or high concentrations of CO_2_ are potentially exacerbated [24]. Because of the individual and interactive effects that temperature, oxygen, and pCO_2_ may have on physiology and other metrics related to fitness and survival [6], [24], [25], knowledge of their covariability is essential for designing OA experiments that adequately characterize biological performance under contemporary relative to future acidified conditions.

Here, we have analyzed a large carbonate chemistry data set from Puget Sound, a large fjord estuary on the U.S. west coast [26]. Puget Sound is a highly altered ecosystem that suffers from multiple stressors stemming from extensive urbanization in the surrounding watershed. Habitat loss, shoreline development, pollution, alteration of freshwater flows, and overfishing are pervasive problems [27]. Despite these impacts, the Sound remains a biologically diverse system that directly supports economically important industries including aquaculture, commercial and recreational fisheries, and tourism [27]. Located on the eastern boundary of the NE Pacific, the Sound is supplied with CO_2_- and nutrient-rich waters that are upwelled onto the continental shelf [26], [28]. Further CO_2_ additions resulting from high rates of respiration act to make Puget Sound a highly vulnerable region for acidification [26]. Due to the reduced carbonate buffering capacity of the seawater, increases in pCO_2_ and reductions in pH resulting from ocean uptake of anthropogenic CO_2_ will occur more rapidly compared to open-ocean waters [26]. Similarly, the saturation state of seawater with respect to biogenic calcium carbonate minerals (calcite and aragonite) will also decline at a faster rate, suggesting that calcifying species may be adversely affected sooner in Puget Sound relative to open ocean populations [26].

We sought to evaluate seasonal and regional variability in carbonate chemistry conditions as well as patterns of covariation with temperature, oxygen, and salinity. Our primary rationale was to provide an evaluation of carbonate chemistry variability in this system as part of a larger effort to develop OA experimental designs with improved ecological relevance. Our second objective was to evaluate the potential range of carbonate chemistry conditions experienced by members of the Puget Sound pelagic food web and how that range may change with additional CO_2_. In particular, we focused on potential simultaneous changes in temperature, oxygen, and carbonate chemistry conditions that are likely to be experienced by pelagic biota undergoing diel vertical migrations across regions and seasons in Puget Sound. In doing so, we aim to draw attention to the more general issue of identifying temperature, oxygen, and carbonate chemistry combinations that reflect plausible control and experimental conditions that are seasonally appropriate and correspond to the habitat occupied by the focal study organism or assemblage. We analyzed water chemistry data collected over three seasons (winter, summer, and fall), which afforded the opportunity to assemble seasonal snapshots of spatial variation in carbonate chemistry. Further, we collected data over two summers (August 2008 and September 2009) and two fall periods (October 2010 and October 2011). The inclusion of summer and fall sampling permitted an evaluation of interannual variation over a particularly dynamic season in Puget Sound. During the transition from late summer to early fall, intrusions of denser oceanic waters at depth result in the upward movement of the most oxygen-depleted waters in southern Hood Canal (a major sub-basin of Puget Sound) and southerly winds over Hood Canal result in episodic, localized upwelling events that bring oxygen-depleted, cooler waters to the surface at the southern end of Hood Canal [29]. We show that carbonate chemistry in Puget Sound covaries strongly with temperature and oxygen, but that the strength and sign of the relationship can change seasonally. Our findings offer insight into patterns of carbonate chemistry variability in this system, and we highlight their implications for the design and interpretation of OA laboratory experiments that simulate conditions representative of this and other coastal systems.

## Materials and Methods

**Figure 1:**
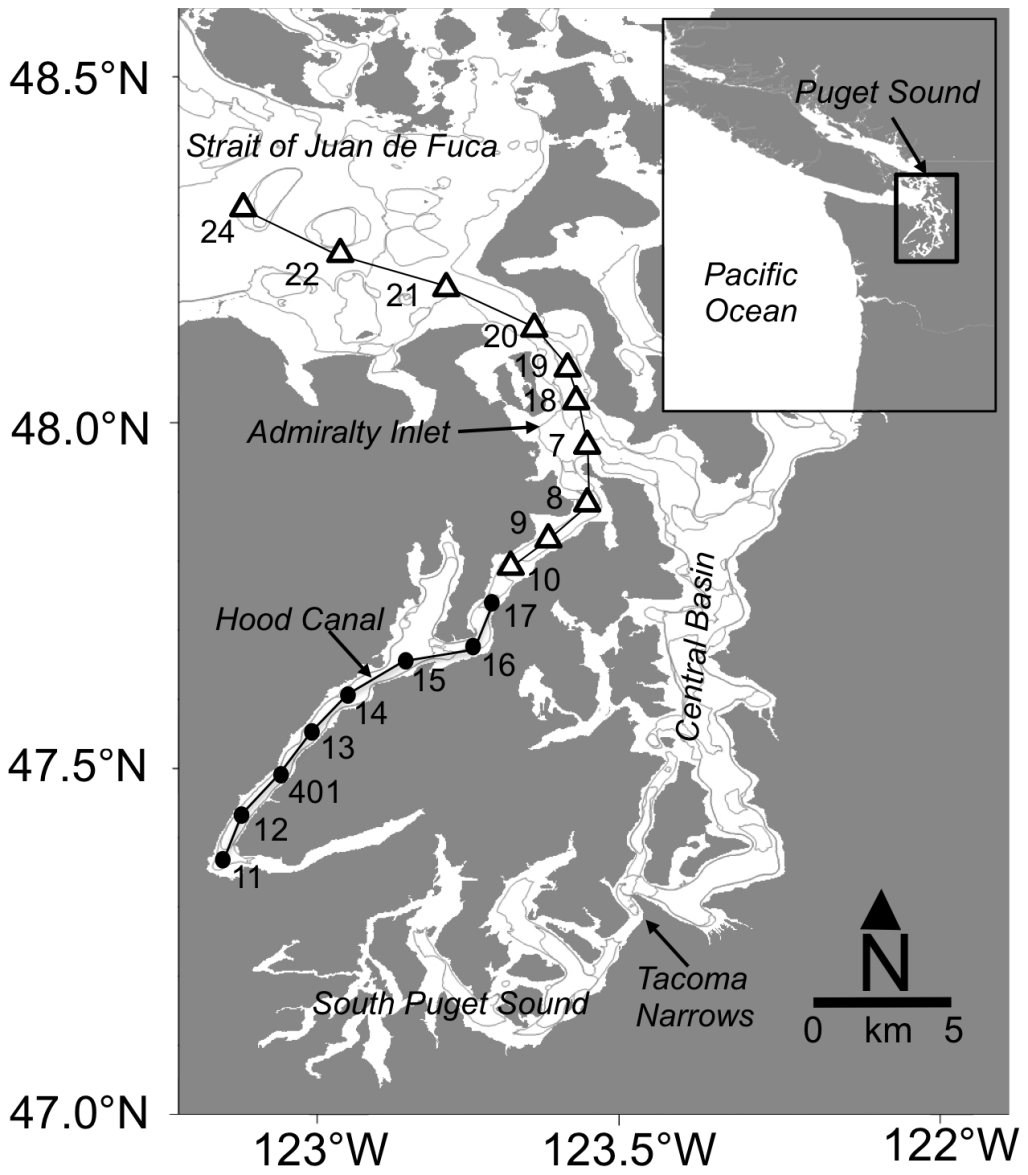
Map of the study area and stations sampled for carbonate chemistry in Hood Canal (denoted by solid circle symbol) and Admiralty Inlet (open triangle) from Puget Sound, Washington, USA. Sampling took place February and August 2008, September 2009, and October 2010 and 2011. Station numbering follows the convention of PRISM cruises (1998–2011, http://nvs.nanoos.org/CruisePrism).

We collected water samples in Puget Sound over five cruises that spanned winter (February 2008), summer (August 2008, September 2009) and fall (October 2010 and 2011; Table 1) as part of the Puget Sound Regional Synthesis Model Program (PRISM) conducted by the University of Washington in collaboration with the Washington Department of Ecology. The summer surveys correspond to conditions prior to the intrusion of denser oceanic waters at depth while the fall surveys capture this process. The full survey includes transect lines with stations in all four major sub-basins of Puget Sound as well as the Strait of Juan de Fuca (Fig. 1). For the current study, we focused on stations south of the sill at the entrance to Hood Canal (herein referred to as ‘Hood Canal’) and those spanning the northern entrance of Admiralty Inlet to northern Central Puget Sound (herein ‘Admiralty Inlet’). The bottom depth of the station near the sill in Hood Canal is ∼100 m while the deepest stations near the center of the basin are ∼170 m. Bottom depths of stations spanning Admiralty Inlet range from 70 m to 160 m. We focused our analysis on these regions because they possess contrasting mixing patterns and were sampled consistently across cruises. All stations were located in Washington State waters and no special permission was required to collect water samples. Field activities did not involve endangered or protected species.

**Table 1:** Dates and stations sampled in Hood Canal and Admiralty Inlet for carbonate chemistry during PRISM cruises from 2008 to 2011.

Cruise	Sampling dates	Stations sampled
Feb. 2008	5 – 7 Feb.	7–22, 24, 401
Aug. 2008	12 – 13 Aug.	7–22, 24, 401
Sep. 2009	30 Sep. – 1 Oct.	7–11, 15–19, 21, 22
Oct. 2010	15 – 30 Oct.	7–22, 24, 401
Oct. 2011	12 – 13 Oct.	7–22, 24, 401

At all stations, full water column profiles of temperature, salinity, and oxygen were obtained using a SeaBird (SBE) 911*plus* conductivity-temperature-density (CTD) instrument equipped with an SBE 43 oxygen sensor (SeaBird Electronics Inc., Bellevue, WA). Data were binned at 0.5-m intervals. To correct oxygen sensor measurements for potential offsets, discrete water samples were analyzed for oxygen by modified Winkler titration [30]. The salinity sensor on the CTD was factory-calibrated annually; the sensor was checked against discrete values collected on the cruise at a subset of stations, analyzed with a Guildline Autosal by the University of Washington, and calibrated, if necessary. Cruise CTD data are available through the NANOOS Visualization Server (http://nvs.nanoos.org/CruisePrism).

Discrete water samples were collected and analyzed for carbonate chemistry following previously published methods [12]. Briefly, water samples were obtained with Niskin-type bottles and analyzed in the laboratory for dissolved inorganic carbon (DIC), total alkalinity (TA), oxygen, and nutrients. DIC was analyzed using coulometric titration [31]–[33]. TA was measured by the potentiometric titration method [33]. Certified Reference Materials (CRMs) were analyzed with both the DIC and TA samples as an independent verification of instrument calibrations [33]. The DIC and TA data have a combined precision and accuracy of ∼1 µmol kg^−1^ and ∼2 µmol kg^−1^, respectively, based on replicate sample analysis and the use of CRMs. Carbonate chemistry measurements from all surveys are currently being prepared for submission and archival at the Carbon Dioxide Information Analysis Center (http://cdiac.ornl.gov/oceans/).

For our analysis, we focused on carbonate chemistry variation as conveyed by changes in pCO_2_ and the saturation state of aragonite (Ω_ar_). We focused on pCO_2_ because this is the most commonly used treatment variable in OA experiments and the parameter directly changed by anthropogenic CO_2_ emissions. We evaluated variation in Ω_ar_ because of its direct relationship to the stability of aragonite material in calcifying species. In supersaturated conditions (Ω_ar_ >1), the formation of aragonite is energetically favored, while dissolution is favored in undersaturated conditions (Ω_ar_ <1). We calculated pCO_2_ and Ω_ar_ using the R library ‘seacarb’ [34] with dissociation constants from Lueker et al. [35]. The pressure effect on the solubility was applied as described from the equation of Mucci [36], incorporating the adjustments to the constants recommended by Millero [37]. Based on the uncertainties in the DIC and TA measurements, uncertainty in the calculated aragonite saturation state and pCO_2_ is approximately 0.015–0.04 and 5–10 µatm, respectively.

We evaluated covariation of both pCO_2_ and Ω_ar_ with temperature, oxygen, and salinity within each survey for Hood Canal and Admiralty Inlet stations using simple linear regression separately for each variable. We evaluated the degree of association based on the variance explained by the regression models (*R* ^2^). To comply with assumptions of normality in the response variable, both pCO_2_ and Ω_ar_ were log_10_-transformed prior to fitting the regression models. The fitted regressions offer information on linear trends between variables but are not suited for the prediction of carbonate chemistry variables using temperature, oxygen concentration, or salinity measurements beyond the region and time period sampled for each cruise.

Next, we sought to visually compare seasonal and spatial patterns in the vertical distribution of pCO_2_ and Ω_ar_. To facilitate comparability, we predicted pCO_2_ and Ω_ar_ across depths at each station using continuous depth profiles of temperature, oxygen, and salinity based on relationships estimated with the discrete carbonate chemistry samples. The predicted pCO_2_ and Ω_ar_ values offered a higher vertical resolution of carbonate chemistry variability at each station than measurements from the discrete water samples alone. In preliminary stages of model development, we first considered multiple regression methods to predict pCO_2_ and Ω_ar_, but significant collinearity in the predictor variables was observed in some regions and time periods (absolute value of Pearson’s correlation coefficient greater than 0.90), which hampered their application [38]. To overcome this issue, we instead utilized partial least squares regression (PLSR) to model pCO_2_ and Ω_ar_. PLSR is a dimension reduction technique that is well-suited to data sets that exhibit multicollinearity among predictor variables [39]. Essentially, latent factors (or component variables) are obtained from linear combinations of the predictor variables. The approach is similar to principal components regression except that in PLSR, component variables are obtained by maximizing the explained variance in the response variables rather than within the predictor variables [39]. We selected the optimal number of components by calculating the root mean square error of prediction (RMSEP) for each component using leave-one-out cross validation. We retained only those components without which the RMSEP did not show a significant decrease [40]. One PLSR model was estimated for pCO_2_ and a second model was estimated for Ω_ar_. We used CTD temperature, salinity and oxygen concentration as predictor variables in both models. However, to predict relationships for all stations across time periods and regions, we also included a categorical variable that denoted the region and cruise from which a sample was obtained along with first-order interactions between the continuous CTD variables and the categorical variable. Doing so permitted the relationships between the carbonate chemistry parameters and temperature, salinity and oxygen to vary between regions and seasons. This was deemed necessary because preliminary examination of the data sets suggested differing relationships between the explanatory and response variables across seasons and regions. As with the simple linear regression models, both pCO_2_ and Ω_ar_ were log_10_-transformed to improve normality in the residual error structure. To evaluate model adequacy, we evaluated the explained variance in the response variable (*R* ^2^), RMSEP, and checked for homogeneity in the residual error structure. We used the fitted model to predict pCO_2_ and Ω_ar_ values across depths using CTD and oxygen sensor measurements and visually compared patterns of carbonate chemistry variation between regions and seasons; we employed PLSR purely as a means to estimate high-resolution vertical profiles of pCO_2_ and Ω_ar_.

We note that although previous studies have developed semi-mechanistic predictive multiple regression models of carbonate chemistry using these variables in the California Current system [15], [23], the application of similar methods to Puget Sound was hampered by several considerations beyond the multi-collinearity we observed among predictor variables. Namely, a key assumption of these models is that the stoichiometry of the CO_2_:O_2_ relationship in constituent water masses is primarily controlled by aerobic respiration acting over the time span since last exposure to the atmosphere. Puget Sound receives waters upwelled onto the continental shelf that are entrained in subsurface flows through the Strait of Juan de Fuca, but that mix to the surface and combine with low salinity, seaward-flowing surface waters due to the presence of shallow sills at Admiralty Inlet and the entrance to Hood Canal [41]. As a result, CO_2_:O_2_ relationships in the Sound reflect a combination of respiration in inflowing upwelled coastal waters, differential diffusion rates of CO_2_ and O_2_ between seawater and the atmosphere, and variable mixing with surface waters where CO_2_:O_2_ relationships are strongly influenced by biological production and freshwater inputs. We expect that semi-mechanistic models may exist with the capacity to estimate at least some carbonate chemistry variables in Puget Sound (e.g. TA), but our primary aim here was to interpolate pCO_2_ and Ω_ar_ values between depths where discrete samples were collected at stations within each survey. The development of models to extrapolate carbonate chemistry parameters across time periods and locations outside our spatial and temporal sampling domain is beyond the scope of the present study but remains an area of active research.

In Puget Sound as elsewhere, many fish and zooplankton, including euphausiids, mysids, copepods, pteropods, and a large variety of fish and invertebrate larvae, undergo diel vertical migrations (DVM) and may swim vertical distances in excess of 50 m in less than an hour [42]–[46]. In stratified waters, diel vertical migrators may pass through water masses with drastically differing temperature, oxygen, and carbonate chemistry conditions [45]. The prevalence of DVM in Puget Sound fish and invertebrates and our need to understand the response of these groups to acidification motivated our interest in describing and comparing potential carbonate chemistry, temperature, and oxygen conditions experienced by diel vertical migrators across seasons and regions. In particular, we evaluated how these variables may change simultaneously as organisms move between near-surface and subsurface waters and compared contemporary carbonate chemistry conditions experienced by diel vertical migrators with those assuming an increase in DIC due to future ocean uptake of anthropogenic CO_2_.

To estimate pCO_2_ and Ω_ar_ under elevated DIC conditions, we used an equilibrium approach based on the thermodynamic equations that describe the carbonate system [5]. From the outset, we recognized the complexity of physical (vertical and horizontal mixing, air-sea CO_2_ fluxes, freshwater inputs) and biological processes (photosynthesis, respiration) that potentially regulate carbonate chemistry conditions in Puget Sound and therefore did not seek to generate precise predictions of pCO_2_ and Ω_ar_ corresponding to specific IPCC scenarios of CO_2_ emissions. Rather, we considered a scenario in which the present-day anthropogenic DIC load in Puget Sound (∼30 µmol kg^−1^; [26]) was quadrupled to 120 µmol kg^−1^. That is, we added 90 µmol kg^−1^ to observed in situ DIC measurements from the discrete water samples, and recalculated the carbonate system to estimate future seawater pCO_2_ and Ω_ar_ (hereafter noted as pCO_2_* and Ω_ar_*). This DIC addition approximates potential anthropogenic CO_2_ loads in mid-latitude surface ocean waters after equilibration with an 788 µatm pCO_2_ atmosphere as predicted for the year 2100 based on the Intergovernmental Panel on Climate Change (IPCC) IS92a ‘continually increasing’ emissions scenario [1], [5]. We derived our DIC increment value from potential changes to inflowing marine waters to Puget Sound because river discharge comprises only 5% of the annual water exchange, and the median water residence time is ∼80 days for Hood Canal and less than 60 days for the remaining sub-basins of Puget Sound [47]. Consequently, inflowing marine waters largely govern temperature, salinity, and nutrient loads in Puget Sound [28], [41], [48] and set initial conditions for carbonate chemistry in bottom waters entering Hood Canal as well [26]. The method carries the implicit assumption that alkalinity, respiration rates, and circulation patterns remain invariant. In summer, strong upwelling winds on the Washington coast supply deep, dense waters to the Sound that are thought to have last been in contact with the atmosphere approximately 50 years prior and that acquired their anthropogenic CO_2_ burden at that time [12]. The anthropogenic CO_2_ burden in Puget Sound can therefore lag behind conditions in open-ocean surface waters by a few decades, and future DIC loads will depend partly on interannual changes to the seasonal upwelling regime. Recognizing these complexities, our application of a uniform increase in DIC across seasons and locations is meant to simplify and facilitate general comparisons of possible future carbonate chemistry variability. As with pCO_2_ and Ω_ar_, we used CTD temperature, salinity, and oxygen values corresponding to pCO_2_* and Ω_ar_* from the discrete water samples to predict pCO_2_* and Ω_ar_* across all depths using PLSR; model selection proceeded in a manner identical to that used for pCO_2_ and Ω_ar_.

Based on typical DVM patterns observed in Puget Sound zooplankton and fish [42], [45], we assumed that zooplankton descended from 3 to 50 m at a rate of 1 m min^−1^ starting at 0600 hr, and ascended from 50 to 3 m beginning at 1800 hr at the same rate. Given this depth pattern over a 24-hour period, we extracted the corresponding CTD data and pCO_2_, pCO_2_*, Ω_ar_, and Ω_ar_* values predicted across depths from each station in the survey to generate time traces. We compared time traces across seasons and between Admiralty Inlet and Hood Canal stations to evaluate potential high-frequency variation in carbonate chemistry resulting from diel movements, explore how carbonate chemistry changes simultaneously with other biologically important variables, and examine how carbonate chemistry may differ under CO_2_-enriched conditions as experienced by diel vertical migrators. To aid comparability, we used the same DVM across seasons and locations, but acknowledge that seasonal, regional, and interspecific variations in DVM may in fact be significant.

## Results

**Figure 2:**
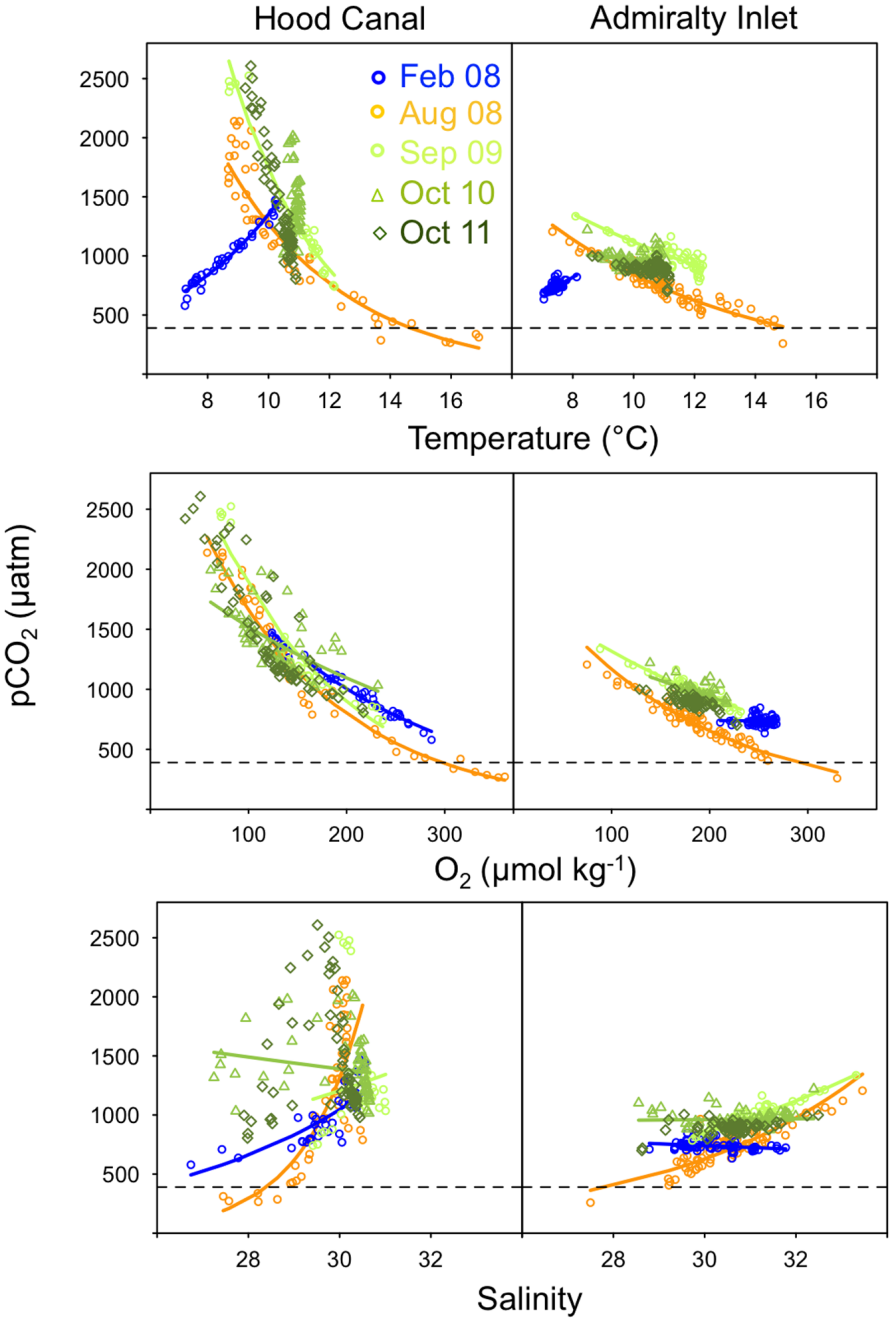
Patterns of covariation between pCO_2_ and temperature (upper), oxygen (middle), and salinity (lower panels) in Hood Canal and Admiralty Inlet, Washington. For reference, the average atmospheric pCO_2_ level (2008–2011 mean: 392 µatm) is noted by a dashed line. Trend lines correspond to relationships fitted using log_10_-transformed pCO_2_.

In Hood Canal, pCO_2_ estimates obtained from discrete water samples ranged from 230 to 2610 µatm, and the majority of water samples exceeded atmospheric pCO_2_ levels (mean atmospheric pCO_2_ from 2008 to 2011: 389 µatm; Fig. 2). Regression intercept and slope coefficients and the explained variance (based on the log_10_-transformed response variables) associated with the trend lines depicted in Fig. 2 are presented in Table S1. The Hood Canal August relationship between pCO_2_ and temperature was negative and spanned the largest range of temperatures (8.5–17°C) relative to the other months surveyed (Fig. 2). In fall, the range of temperatures observed in both years was narrower relative to summer (∼9–12°C; Fig. 2). Temperatures in winter ranged from 8 to 11°C in Hood Canal but showed the opposite relationship with pCO_2_ as observed in the summer and fall (Fig. 2). Similar temperature-pCO_2_ relationships were evident for Admiralty Inlet, but for all cruises the range of temperatures and pCO_2_ levels were narrower. In general, variation in pCO_2_ was more tightly associated with temperature in Hood Canal relative to Admiralty Inlet based on the higher *R* ^2^ values observed in Hood Canal across all seasons (Table S1).

Negative relationships between pCO_2_ and oxygen were evident in all seasons and years in both Hood Canal and Admiralty Inlet with the exception of Admiralty Inlet in February (Fig. 2, Table S1). As with temperature, the larger range of O_2_ values were found in Hood Canal relative to Admiralty Inlet (Fig. 2). In general, the negative relationship between pCO_2_ and O_2_ was stronger in Hood Canal than Admiralty Inlet based on *R* ^2^ values (Table S1). Salinities, in contrast, spanned a relatively narrow range of values (∼27 to 33; Fig. 2). In both regions, relationships between pCO_2_ and salinity were positive in August. In the remaining seasons, pCO_2_ and salinity relationships were weaker. Overall, pCO_2_ was less tightly associated with salinity relative to temperature and oxygen across all seasons and both regions (Fig. 2; Table S1).

**Figure 3:**
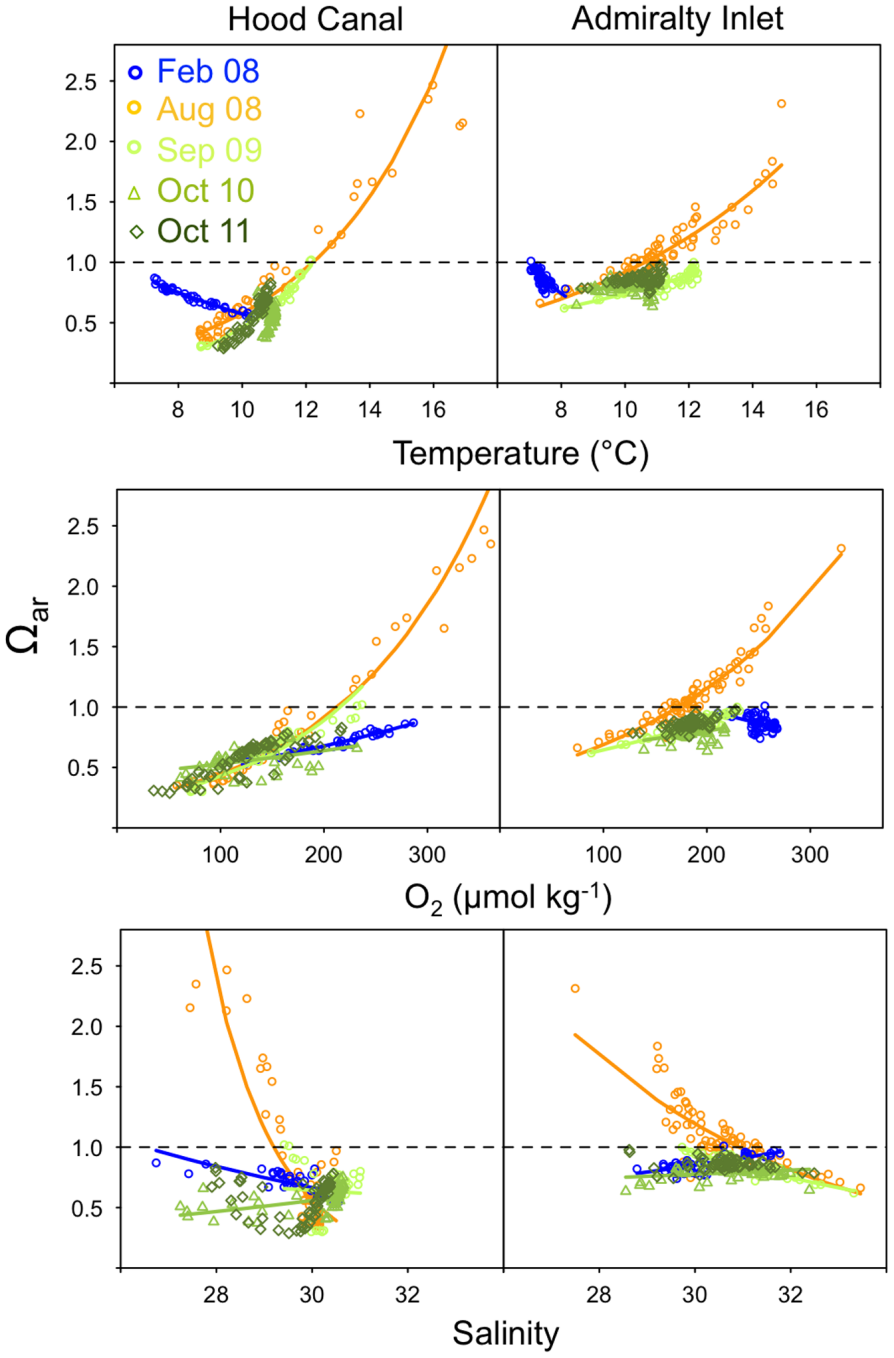
Patterns of covariation between aragonite saturation state (Ω_ar_) and temperature (upper), oxygen (middle), and salinity (lower panels) in Hood Canal and Admiralty Inlet, Washington. Values below 1 (indicated by dashed line) correspond to corrosive conditions, those above are supersaturated with respect to aragonite. Trend lines correspond to relationships fitted using log_10_-transformed Ω_ar_.

As expected, patterns of Ω_ar_ were nearly inverse to those observed for pCO_2_ (Fig. 3; Table S1). With the exception of August, when *in situ* Ω_ar_ values ranged from 0.2 to 2.8, Ω_ar_ values were ∼1 or lower in both Hood Canal and Admiralty Inlet in all remaining months (Fig. 3). Similar to pCO_2_, the range of Ω_ar_ values was highest in Hood Canal and lowest in Admiralty Inlet (Fig. 3). Patterns of association between Ω_ar_ and temperature, oxygen, and salinity based on *R* ^2^ values were similar to those of pCO_2_ between regions and across seasons (Table S1).

**Figure 4:**
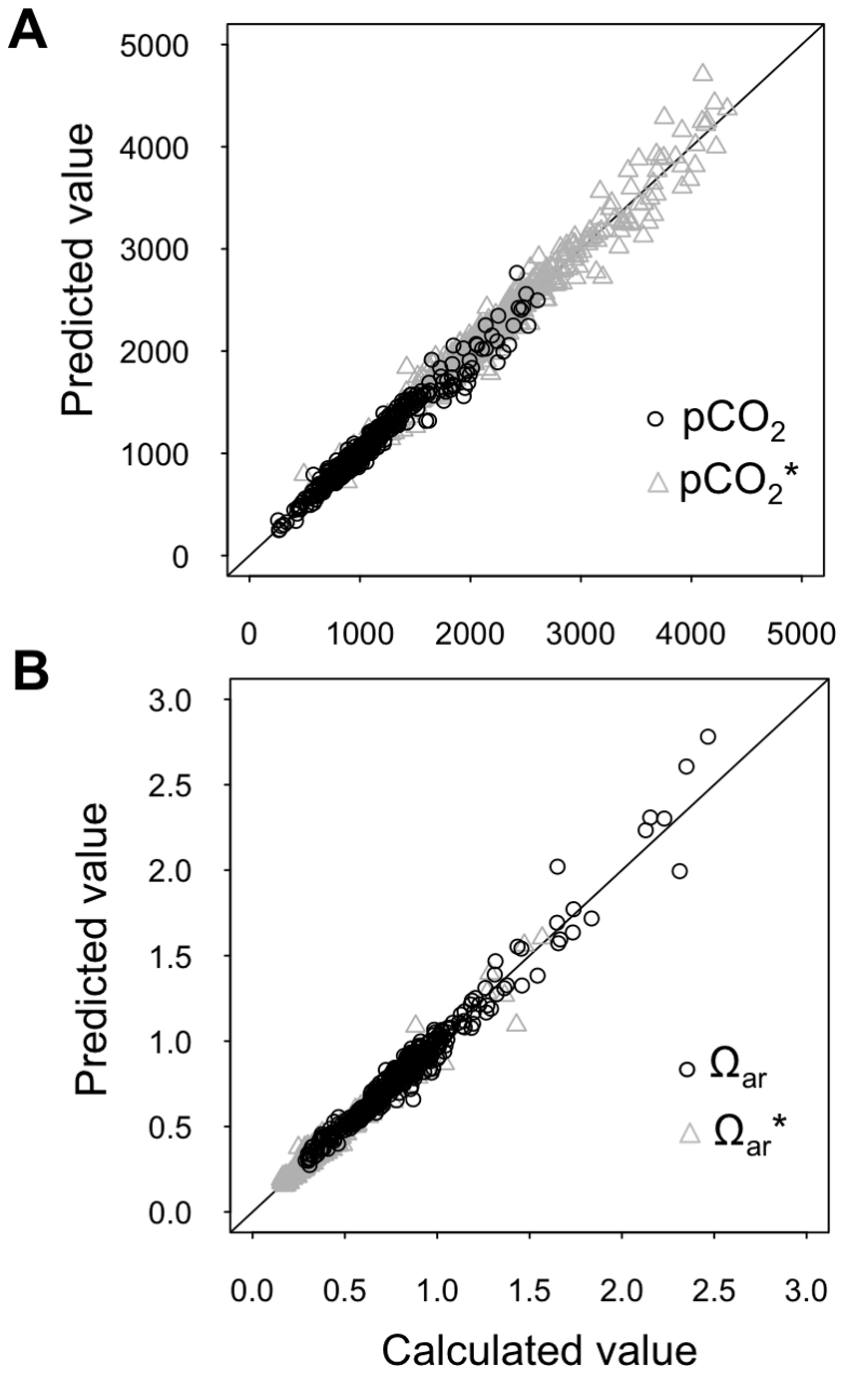
(a) Predicted versus calculated pCO_2_ and pCO_2_* and (b) Ω_ar_ and Ω_ar_* based on partial least squares regression models for Puget Sound water samples obtained from 2008 to 2011. pCO_2_* and Ω_ar_* values correspond to estimates based on an the addition of 90 µmol kg^−1^ DIC across all samples.

Partial least square regression models of pCO_2_ and Ω_ar_ explained greater than 95 and 94% of variance in each survey, respectively (Fig. 4). RMSEPs (based on the original response variable scale) ranged between 4 and 50 µatm and 0.02 and 0.05, for pCO_2_ and Ω_ar_, respectively. The model fits suggested a sufficient level of predictive accuracy for estimating values across depths based on CTD depth profiles of oxygen, temperature, and salinity. We predicted pCO_2_ and Ω_ar_ across depths and examined temperature and oxygen depth profiles as well, but did not evaluate salinity due to the narrow range of values observed in the survey.

**Figure 5:**
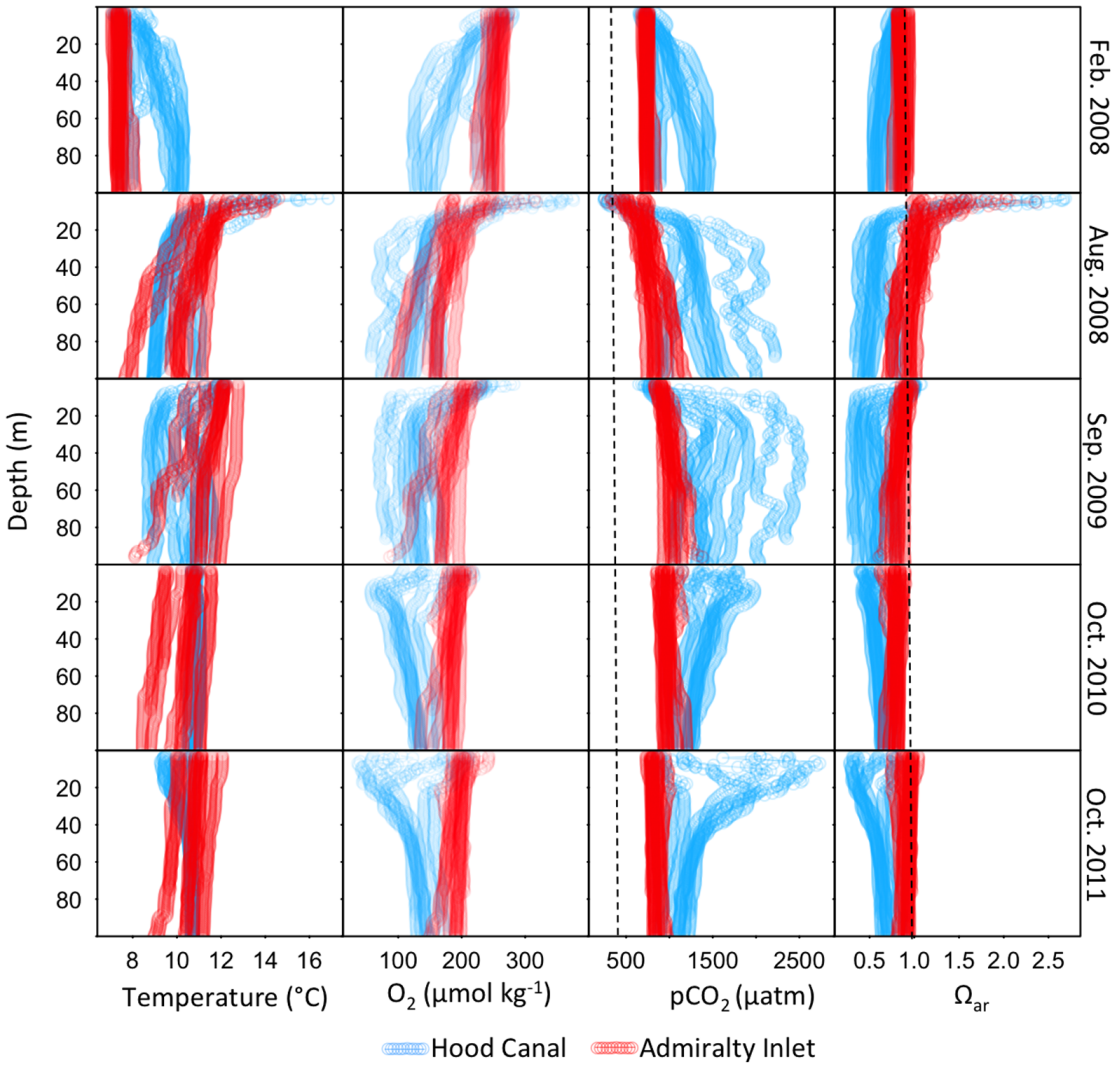
Temperature and oxygen CTD depth profiles at stations sampled in Hood Canal and Admiralty Inlet. pCO_2_ and Ω_ar_ values are model-based estimates obtained using partial least squares regressions. To aid interpretation, vertical dashed lines are overlaid denoting the average atmospheric pCO_2_ (2008 to 2011 mean: 392 µatm) and the equilibrium aragonite saturation state (Ω_ar_  = 1). Profiles are truncated at 100 m to aid comparability.

Overall, seawater pCO_2_ values were above mean atmospheric levels (392 µatm) at all depths and across surveys with the exception of shallow waters (< 15 m depth) at several stations sampled in August 2008 (Fig. 5). Waters were also undersaturated with respect to aragonite (Ω_ar_<1) at all depths sampled in February 2008 and October 2010 and 2011 (Fig. 5). Ω_ar_ in near-surface waters (< 15 m) at some stations in Hood Canal in August 2008 and September 2009, however, were supersaturated (Ω_ar_ >1), while several stations at Admiralty Inlet sampled in August 2008 were supersaturated throughout the water column (Fig. 5).

In general, large differences in vertical gradients in carbonate chemistry, temperature, and oxygen conditions were apparent across seasons and regions. In February 2008, CTD depth profiles of temperature and oxygen and predicted values of pCO_2_ and Ω_ar_ indicated waters in Admiralty Inlet were well-mixed, and showed little vertical or inter-station variability (Fig. 5). In Hood Canal, several stations were also well-mixed, though vertical gradients were evident at several stations: deep waters were warmer, oxygen depleted, rich in pCO_2_, and exhibited lower Ω_ar_ values relative to surface waters (Fig. 5). In August 2008 and September 2009, the water column was strongly stratified at several stations in Hood Canal, with warm, oxygen-rich, low pCO_2_, supersaturated waters occurring in near-surface waters. At depth, the opposite conditions prevailed. Variation in water conditions was comparatively lower at Admiralty Inlet both across stations and vertically (Fig. 5).

Stratification was again low at Admiralty Inlet stations in October 2010 and 2011 with near-uniform temperature, oxygen, and carbonate chemistry conditions throughout the water column and little variability between stations. However, in Hood Canal oxygen-poor, CO_2_-rich, and low Ω_ar_ waters occurred in the top 50 m of the water column, while the opposite conditions were observed at depth (Fig. 5). Hypoxic conditions (oxygen concentrations less than ∼60 µmol kg^−1^) were observed in waters at depths shallower than 20 m at stations in southern Hood Canal in October 2011. Across all five surveys, the highest and lowest estimated pCO_2_ and Ω_ar_ values, respectively, were observed at these same stations and depths: estimates of pCO_2_ exceeded 2400 and Ω_ar_ values were less 0.4 (Fig. 5).

**Figure 6:**
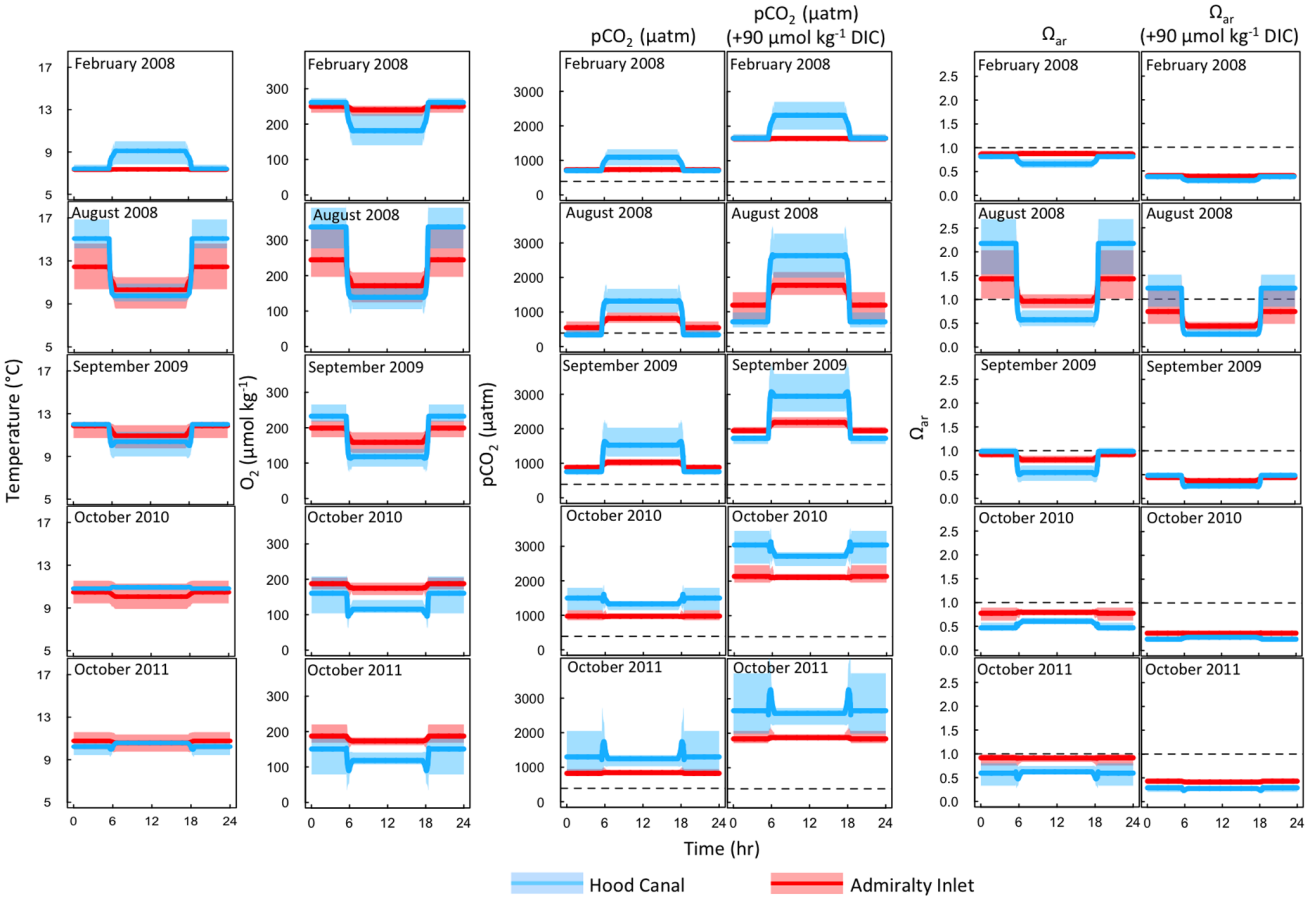
Time traces of temperature, oxygen, pCO_2_ and Ω_ar_ conditions potentially experienced by hypothetical diel vertical migrators at stations sampled in Puget Sound, Washington, U.S.A. Organisms ascend from 50 to 3^−1^ beginning at 1800 hr and descend back to 50 m at 0600 hr. Colored bands correspond to maximum and minimum pCO_2_ and Ω_ar_ values and pCO_2_* and Ω_ar_* as estimated assuming a 90 µmol kg^−1^ increase in DIC in Hood Canal and Admiralty Inlet stations. The solid lines correspond to mean values. For comparison, atmospheric pCO_2_ (2008–2011 average: 392 µatm) is indicated by a dashed line. Ω_ar_ values below 1 (denoted by dashed line) indicate undersaturated conditions with respect to aragonite.

Time traces of temperature, oxygen, pCO_2_, and Ω_ar_ concentrations corresponding to conditions experienced by hypothetical diel vertical migrators are depicted in Fig. 6. As suggested by the depth profiles, diel vertical migrators occupying the sampled stations likely experience simultaneous and abrupt changes in temperature, oxygen, and carbonate chemistry conditions in strongly stratified locations, and these were most prevalent in Hood Canal and Admiralty Inlet in August 2008 and September 2009 (Fig. 6). Time traces in August 2008 in Hood Canal, for instance, suggest that mean temperature, oxygen concentration, Ω_ar_, and pCO_2_ experienced by diel vertical migrators in near-surface waters (3 m) at night and deep waters (50 m) during the day differed by 5.1°C, 214 µmol kg^−1^, 1.68, and 1100 µatm, respectively (Fig. 6). Importantly, the ranges across stations in temperature, oxygen, and carbonate chemistry conditions in both surface and deep waters were also notable during this time period in Hood Canal. The larger range in carbonate chemistry variables occurred in deep relative to near-surface waters: minimum and maximum pCO_2_ values in near-surface waters were estimated to be 250 and 480 µatm and in deep waters values were 980 and 2030 µatm, respectively. Ω_ar_ ranged from 1.52 to 2.68 in near-surface waters and 0.37 to 0.77 in deep waters (Fig. 6).

In all seasons and regions, pCO_2_* values were not only higher relative to pCO_2_, but the difference between near-surface and deep-water values increased (Fig. 6). In Hood Canal August 2008, the mean difference between near-surface and deep-water pCO_2_ widened from 1100 to 2090 µatm with the uniform addition of DIC (Fig. 6). Correspondingly, Ω_ar_ decreased across all depths as well, but the mean difference between near-surface and deep water was lower (1.68 vs. 0.98) relative to Ω_ar_, as the decline in the carbonate ion concentration slows as it approaches zero (Fig. 6).

In contrast to August 2008 and September 2009, the mean differences in temperature, oxygen, and carbonate chemistry variables between near-surface and deep waters were lower in October 2010 and 2011 (Fig. 6). However, the upward displacement of the waters with the lowest oxygen concentrations toward the surface in Hood Canal resulted in time traces in which diel vertical migrators encountered higher pCO_2_ and lower Ω_ar_ during the night rather than during the day (Fig. 6). Further, the range in pCO_2_ and Ω_ar_ conditions in Hood Canal was larger among stations in near-surface relative to deep waters, and these ranges increased and decreased, respectively, with an increase in DIC (Fig. 6).

Overall, the difference in near-surface and deep temperatures, oxygen concentrations, and carbonate chemistry conditions experienced by diel vertical migrators in February 2008 were lower than in the remaining surveys (Fig. 6). Similar to August 2008 and September 2009, oxygen concentrations and Ω_ar_ were lower and pCO_2_ higher for diel vertical migrators at depth during the day in Hood Canal, but unlike the remaining months surveyed the mean temperature was warmer by 1.7°C relative to near-surface waters experienced during the night. Further, the potential range of water conditions experienced by diel vertical migrators at a given depth across stations was narrower than observed in the remaining surveys (Fig. 6).

## Discussion

Our study expands on previous research on Puget Sound carbonate chemistry that noted waters greatly enriched in CO_2_ relative to air-sea equilibration conditions in the winter and summer [26]. Here, we show that CO_2_-enriched conditions persist through the fall. We also show that carbonate chemistry conditions covary with temperature and oxygen but that the direction and strength of these correlations change with season and between nearby regions characterized by differing levels of water mass mixing. Our findings suggest that fish and invertebrates that exhibit DVM behavior in stratified locations are likely to experience large, rapid changes in ambient carbonate chemistry conditions over the course of a single day during the summer and early fall months. Further, we show through simple calculations that a uniform increase in DIC not only elevates pCO_2_ and lowers Ω_ar_, but also widens the absolute difference in these values between near-surface and subsurface waters. Together, our findings highlight the physicochemical complexity of coastal systems and the importance such data hold for the design of ecologically relevant OA experiments.

Our observations in September 2009 and October 2010 and 2011 add an additional layer of information to our general understanding of seasonal variability in carbonate chemistry in Hood Canal. Due to sluggish circulation and the availability of nutrients in inflowing marine and terrestrial waters, hypoxic conditions commonly occur in Hood Canal during the summer months [49]. As evidenced by oxygen and pCO_2_ depth profiles in August 2008 and September 2009, aerobic respiration simultaneously draws down O_2_ at depth and increases pCO_2_. However, we show that bottom-water intrusions into Puget Sound and Hood Canal during October 2010 and 2011 resulted in the upward movement of low-oxygen waters as observed previously [45] but that these near-surface waters were CO_2_-replete and exhibited the most extreme pCO_2_ and Ω_ar_ values observed across all surveys. Fall bottom-water intrusions into the Sound occur during neap tides and their onset is influenced by storm-related fluctuations in the horizontal density gradient spanning the double-silled entrance at Admiralty Inlet [50], [51]. Although such intrusions play a critical role in the renewal of deep water layers in Puget Sound, they also result in the surfacing of low oxygen, high pCO_2_ waters which, if upwelled suddenly to the surface due to favorable southerly winds, can result in extensive fish kills [29]. Our observations suggest that organisms that reside in shallow waters to avoid low-oxygen, CO_2_-rich waters at depth in the summer may be unable to avoid exposure at some locations in Hood Canal as these waters circulate upward in the fall. Combined, our carbonate chemistry data set indicates that pCO_2_ values are spatially and seasonally heterogeneous in Puget Sound as in other estuarine systems [52] and further underscores the need for researchers to consider natural variability in the design and interpretation of OA experiments.

Importantly, we show that carbonate chemistry conditions also covary with temperature and oxygen but that the strength and slope of the relationships change with season. Failure to consider covariation in carbonate chemistry with temperature or oxygen may result in experimental designs with ‘control’ treatments that have little correspondence to conditions that animals may have adapted to in nature and ‘acidified’ treatments that do not account for naturally high pCO_2_ levels in many estuarine and coastal waters. For instance, strong negative relationships between pCO_2_ and temperature in Hood Canal for August 2008 suggest that appropriate control pCO_2_ values for experiments on a widely distributed animal from this region would depend on the temperatures considered in the experiment. Temperatures of 10 and 15°C occur within the top 50 m of the water column and have corresponding control pCO_2_ values of 1270 and 360 µatm, respectively, based on the linear regression between pCO_2_ and temperature. Assuming a uniform increase in 90 µmol kg^−1^ in DIC, the respective acidified treatments are 2150 and 760 µatm, again based on the regression between pCO_2_* and temperature. In contrast, experimental controls based on winter Hood Canal conditions that use temperatures of 8 and 10°C would have pCO_2_ values corresponding to 850 and 1360 and pCO_2_* values of 1380 and 1930 µatm, respectively. Although there is increasing recognition that researchers should consider control treatments that reflect carbonate chemistry conditions experienced by the organism under study [11], [18], [21], little attention has been given to understanding patterns of carbonate chemistry covariability with temperature and oxygen. Given the combined effect environmental temperature, oxygen, and pCO_2_ have on metabolic scope [53] and evidence indicating non-additive, interactive effects of these variables in ecological studies [6], [54], we recommend care be taken in selecting appropriate values that correspond to those likely to be experienced by organisms in the field. Doing so may facilitate inferences from experimental results to population-level effects.

High-resolution carbonate chemistry time series have revealed considerable diel variability in coral reef [55], [56], kelp forest [10], [57], coastal upwelling [58], and shallow estuarine systems [59] that is primarily driven by oscillations in photosynthesis and respiration rates. For animals that undergo DVM in vertically heterogeneous regions, however, exposure to water masses with differing carbonate chemistry, temperature, and oxygen characteristics is also possible but has received only limited consideration in the context of OA [60]. In Puget Sound, DVM behavior is common in a diversity of fish and invertebrate species, including zooplankton occurring in stratified regions of Hood Canal [42], [45]. Developing experiments with temporally dynamic carbonate chemistry, temperature, and oxygen conditions that mimic the experience of organisms in the wild may offer more accurate baselines upon which responses to acidified treatments can be compared. Our hypothetical time traces offer a basis for developing such experimental designs and offer important insights. Foremost, we show that during the summer in particular, temperature, oxygen, and carbonate chemistry conditions simultaneously covary with depth, a consideration that has previously been overlooked in OA experiments focused on diel migrating animals from stratified waters [60]. Organismal performance (e.g., growth) may potentially improve in dynamic pCO_2_ treatments that emulate natural diel oscillations in carbonate chemistry relative to static pCO_2_ treatments [61], but we are unaware of studies that have simultaneously varied pCO_2_, temperature, and oxygen to replicate the full suite of environmental changes associated with DVM. The technical challenges associated with implementing dynamic, multi-stressor experiments are considerable but OA experimental facilities are becoming increasingly sophisticated and such systems are currently under development at several academic and government laboratories.

Our recalculation of the carbonate system to generate estimates of potential future pCO_2_ and Ω_ar_ values experienced by animals undergoing DVM showed that pCO_2_ and Ω_ar_ increased and decreased, respectively, at all depths as expected with an increased CO_2_ load. However, the potential difference in pCO_2_ values between near-surface and deep waters increased relative to contemporary observations, while the potential surface-to-deep difference for Ω_ar_ decreased. This pattern results from nonlinearity in the carbonate system at near-surface ocean pH values: as DIC is added in the form of CO_2_ (either from anthropogenic CO_2_ emissions or respiration), the carbonate ion concentration of seawater declines, while the CO_2_ concentration and pCO_2_ forms of inorganic carbon increase exponentially [62], [63]. In waters already burdened with high concentrations of CO_2_ due to respiration, an increase in DIC results in an increase (decrease) in pCO_2_ (Ω_ar_) that will be larger (smaller) relative to less burdened waters [62], [64]. Our estimates of potential change in carbonate chemistry variability as experienced by DVM fauna are qualitatively similar to simulations in other studies that have shown an amplification of natural variability in carbonate chemistry associated with increased DIC [65]. These findings suggest more broadly that changes in the mean as well as the variance of pCO_2_ will accompany increased DIC loadings. The influence of changes to pCO_2_ variance on organisms is poorly understood and warrants further attention.

In addition, we show that although spatial and vertical variation in carbonate chemistry is considerable in Puget Sound, additional DIC may place pCO_2_ and Ω_ar_ at most stations and depths outside the range of values observed presently across depths and stations. This possibility was most evident in winter where there was no overlap between present-day and future pCO_2_ and Ω_ar_ as experienced by fauna undergoing DVM. During winter, the water column is less stratified and conditions are more uniform across stations and depths [41]. Consequently, low pCO_2_, supersaturated refugia are absent and carbonate chemistry is relatively homogenous. In contrast, some near-surface waters in August remained supersaturated (Ω_ar_) and the pCO_2_ at levels low, despite the DIC addition. These same near-surface waters initially possessed low DIC concentrations due to biological drawdown through photosynthesis and thus higher buffering capacity.

The general seasonal patterns observed suggest that species with CO_2_-sensitive life history stages that occur in winter are even less likely to find depths or regions with favorably low pCO_2_ conditions in the future. For species with CO_2_-sensitive life history stages that occur in summer, the potential for carbonate chemistry refugia persisting in the future may be better because high rates of primary production can locally drive pCO_2_ levels below air-sea equilibrium in the surface layer, though the benefits of such refugia depend on the suitability of other biological variables and their persistence in time and space. Our analysis also suggests that CO_2_-sensitive species may be more fully excluded from regions such as Admiralty Inlet that are well-mixed and therefore lack low pCO_2_ refugia at most depths and stations relative to more heterogeneous regions like Hood Canal.

We limited our ocean acidification scenario to a uniform change in DIC. Our estimates therefore do not account for processes that may further alter carbonate chemistry and the vertical and spatial distribution of CO_2_, which may include climate variables (upwelling winds, circulation patterns, river discharge), changes in watershed use, eutrophication [9], and atmospheric deposition of nitrogen and sulfur from shipping vessels [66] or urban pollution [67]. Processes that reduce salinity (and thus TA) or that increase respiration (and DIC) increase pCO_2_ [64], while warming directly affects carbonate system thermodynamics, resulting in solubility-related increases in pCO_2_, but more importantly influences ecosystem metabolism, which strongly affects carbonate chemistry [68]. Biogeochemical models of coastal and estuarine systems are increasingly utilized to identify the potential processes that dampen or exacerbate acidification [69], [70] and may help clarify the contribution of climate variability and different human activities to future changes in Puget Sound’s carbonate chemistry. We also note that our data set does not permit an evaluation of diurnal carbonate chemistry patterns, which can be pronounced in coastal surface waters, and is limited to stations in open-water habitats. We still lack information on shallower habitats such as mudflats, eelgrass meadows, pocket estuaries, and oyster beds, which are important components of the Puget Sound ecosystem. We recommend targeted sampling in these key habitats over seasonal cycles and the development of long-term monitoring programs in order to better identify baseline carbonate chemistry variability and to facilitate the attribution of the contribution of anthropogenic CO_2_.

Puget Sound receives waters upwelled along the Pacific Coast that are already burdened with anthropogenic CO_2_ [26], and concentrations will continue to increase over the proceeding decades regardless of possible changes to atmospheric pCO_2_. This is because upwelled waters were last in contact with the atmosphere ∼50 years prior to surfacing and therefore carry CO_2_ burdens that reflect air-sea equilibration at that time [12]. Consequently, waters already in transit to upwelling centers on the Pacific coast near Puget Sound carry increasing concentrations of anthropogenic CO_2_ [12]. Although we are unable to stem the flow of CO_2_-enriched marine waters into Puget Sound, understanding how human-caused impacts (e.g., eutrophication, pollution, and altered river flows) exacerbate or buffer against the effects of ocean acidification on biota may help identify actions regional and local governments can take to reduce the effects of OA. A key uncertainty facing coastal ecosystem management, however, lies in the response of organisms and communities to ocean acidification and its interactions with other stressors [2]. Fundamental to addressing this uncertainty is the design of manipulative experiments in which control water conditions are representative of the system under study. Our analyses offer insight into seasonal patterns of covariation in carbonate chemistry with temperature and oxygen, and we suggest that such considerations are needed in order to draw useful ecological inferences from manipulative experiments. Given the potential for synergistic interactions between temperature, oxygen and carbonate chemistry on organismal physiology [24], developing a baseline understanding of how these variables naturally covary can facilitate the development of experiments with more relevance to questions regarding ocean acidification.

## Supporting Information

Table S1 Estimated intercept and slope coefficients and explained variance (*R* ^2^) corresponding to linear regression models relating log_10_-transformed pCO_2_ and Ω_ar_ to temperature, oxygen, and salinity. Slope coefficients that differ from zero are indicated by asterisks. Slopes with p-values <0.05, <0.01, and <0.001 correspond to *, **, and ***, respectively.
(XLSX)
